# Evaluating feasibility of functional near-infrared spectroscopy in dolphins

**DOI:** 10.1117/1.JBO.28.7.075001

**Published:** 2023-07-13

**Authors:** Alexander Ruesch, Deepshikha Acharya, Eli Bulger, Jiaming Cao, J. Christopher McKnight, Mercy Manley, Andreas Fahlman, Barbara G. Shinn-Cunningham, Jana M. Kainerstorfer

**Affiliations:** a Carnegie Mellon University, Neuroscience Institute, Pittsburgh, Pennsylvania, United States; b Carnegie Mellon University, Department of Biomedical Engineering, Pittsburgh, Pennsylvania, United States; c University of St. Andrews, Sea Mammal Research Unit, Scotland, United Kingdom; d Siegfried & Roy’s Secret Garden and Dolphin Habitat, The Mirage Hotel and Casino, Las Vegas, Nevada, United States; e Fundación Oceanogràfic de la Comunitat Valenciana, Valencia, Spain; f Global Diving Research SL., Valencia, Spain; g Kolmården Wildlife Park, Kolmården, Sweden

**Keywords:** marine mammals, functional near-infrared spectroscopy, tissue optical properties, dolphin

## Abstract

**Significance:**

Using functional near-infrared spectroscopy (fNIRS) in bottlenose dolphins (*Tursiops truncatus*) could help to understand how echolocating animals perceive their environment and how they focus on specific auditory objects, such as fish, in noisy marine settings.

**Aim:**

To test the feasibility of near-infrared spectroscopy (NIRS) in medium-sized marine mammals, such as dolphins, we modeled the light propagation with computational tools to determine the wavelengths, optode locations, and separation distances that maximize sensitivity to brain tissue.

**Approach:**

Using frequency-domain NIRS, we measured the absorption and reduced scattering coefficient of dolphin sculp. We assigned muscle, bone, and brain optical properties from the literature and modeled light propagation in a spatially accurate and biologically relevant model of a dolphin head, using finite-element modeling. We assessed tissue sensitivities for a range of wavelengths (600 to 1700 nm), source–detector distances (50 to 120 mm), and animal sizes (juvenile model 25% smaller than adult).

**Results:**

We found that the wavelengths most suitable for imaging the brain fell into two ranges: 700 to 900 nm and 1100 to 1150 nm. The optimal location for brain sensing positioned the center point between source and detector 30 to 50 mm caudal of the blowhole and at an angle 45 deg to 90 deg lateral off the midsagittal plane. Brain tissue sensitivity comparable to human measurements appears achievable only for smaller animals, such as juvenile bottlenose dolphins or smaller species of cetaceans, such as porpoises, or with source–detector separations ≫100  mm in adult dolphins.

**Conclusions:**

Brain measurements in juvenile or subadult dolphins, or smaller dolphin species, may be possible using specialized fNIRS devices that support optode separations of >100  mm. We speculate that many measurement repetitions will be required to overcome hemodynamic signals originating predominantly from the muscle layer above the skull. NIRS measurements of muscle tissue are feasible today with source–detector separations of 50 mm, or even less.

## 1 Introduction

Toothed whales (odontocetes), such as dolphins and porpoises, use echolocation to hunt prey, navigate, and communicate under water.^1^ Their brains process information encoded in echoes to extract subtle spatiotemporal cues that allow perception of auditory objects,^2^ such as prey items, obstacles, or conspecifics. In most mammals, attention is known to modulate what information the brain processes in complex settings with multiple competing sources.^3^^,^^4^ Auditory object forming and attention modulation remains a challenge in humans, especially in crowded environments with multiple sources of sound.^5^ Understanding how the brain modulates attention in echolocating animals, which rely heavily on sound as a dominant sensor modality in a sensory-challenging and dynamic environment, is therefore offering a unique population to understand this cognitive process.

Attempts to measure brain activity in dolphins have been made previously, using modalities such as functional magnetic resonance imaging (fMRI),^6^^,^^7^ measuring hemodynamic responses to neuronal activity, or electroencephalography (EEG),^8^^,^^9^ which measures the electrical field created by coordinated activity of neuronal clusters. fMRI has a high spatial resolution and can therefore distinguish brain activity in neighboring cortical locations but requires aquatic mammals to be removed from the water and transported to a nearby MRI facility. This can limit the variety and effectiveness of conscious attention-based tasks for the animal to perform. Meanwhile, EEG has a high temporal resolution, can be made portable, and can be used underwater if electrodes are insulated from saltwater. However, EEG offers only limited differentiation of brain regions due to a lower spatial resolution.

Given the specific limitations of current methodologies, near-infrared spectroscopy (NIRS) may be an additional tool that could improve capacity for measurement of cortical activity in cetaceans. Functional NIRS (fNIRS) is an imaging modality that, like fMRI, measures the hemodynamic response to neuronal activity utilizing the differences in light absorption between oxygenated and deoxygenated blood. Light of specific red to near-infrared wavelengths (typically 700 to 900 nm for human brain measurements) is shown onto the scalp from where it is scattered diffusely into the tissue, reaching the cortex of the brain. A fraction of this diffuse light scatters back to a photo-detector placed on the skin multiple centimeters away from the source. Changes in light intensity can then be correlated to changes in hemoglobin concentrations, using the modified Beer–Lambert’s law.^10^

NIRS devices can be made portable and water resistant,^11^^–^^13^ and high-density fNIRS has previously been shown to reach fMRI-comparable spatial resolution in human measurements if tomographic reconstruction using multiple spatially separated measurements is performed.^14^ However, the anatomical and morphological differences of dolphins pose key challenges to the function of NIRS. Challenges such as light absorption in muscle and subcutaneous fat (blubber) tissue above the dolphin brain need to be addressed. The primary challenge of using fNIRS in dolphins is the penetration depth required to reach the brain. Although the skin to cortex distance in human adults is ∼10 to 20 mm,^15^ the thicker skin and additional muscle and blubber layer seen in adult bottlenose dolphins increases this distance to 40 mm and more.

Approaches to improve the optical penetration depth of NIRS have been reported before. For example, work on breast cancer detection has utilized anatomical reconstruction based on many source and detector combinations (called diffuse optical tomography), benefited from a cylindrical shape of the target tissue^16^ or compression of the tissue^17^^–^^19^ to overcome penetration depth challenges. In transabdominal fetal pulse-oximetry, large source–detector separations in combination with higher light intensities are utilized, as well as more sensitive detectors and advanced signal processing.^20^^–^^22^ To evaluate the utility of fNIRS for brain imaging and NIRS for physiological measurements in dolphins, we simulated light propagation in a 3D computational dolphin model and determined optimal hardware parameters. This required the creation of an anatomical dolphin model with realistic optical properties of light absorption, scattering, and refraction. Optical properties for terrestrial mammals can be found in the literature, such as Jacques,^23^ for bone, muscle, fat, skin, and other tissues. However, optical properties for marine mammals, especially with respect to their unique blubber tissue, have not been sufficiently characterized.

Here we use optical properties of dolphin blubber and skin measured with frequency-domain NIRS and simulate results of light propagation in a 3D model of a dolphin. We also show expected brain tissue sensitivity (TS) as a function of wavelengths, source–detector distances, and optode placement. Our results indicate that functional brain measurements are challenging and would require specialized equipment. We therefore contrasted the findings in brain TS to muscle TS as an alternative application for NIRS in marine mammal physiology.

## 2 Simulation of Photon Travel in Dolphin Tissue

### 2.1 Optical Properties of Dolphin Tissue

We created a dolphin head model that was divided into four tissue types: “brain,” “bone,” “blubber and skin” (sculp), and “muscle and other tissues.” Optical properties of absorption coefficient ($\mu_{a}$), reduced scattering coefficient ($\mu_{s}^{\prime }$), and the refraction index ($r_{i}$) were defined for each tissue type. We calculated $\mu_{a}$ and $\mu_{s}^{\prime }$ as a function of wavelength using literature values reported by Jacques^24^ for brain, bone, and muscle tissues. For the marine mammal–specific blubber tissue, optical properties were measured using a combination of frequency domain NIRS (FD-NIRS) and broadband NIRS.

For the optical properties of blubber and skin, we measured bottlenose dolphins (*Tursiops truncatus)* at the Fundación Oceanogràfic de la Comunitat Valenciana, Valencia, Spain; the Siegfried and Roy’s Secret Garden and Dolphin Habitat at the Mirage Las Vegas Hotel & Casino, Las Vegas, Nevada, United States; and Dolphin Quest Oahu, Honolulu, Hawaii, United States. All research procedures were approved by the Oceanogràfic Animal Care & Welfare Committee at the Fundación Oceanogràfic Valencia, Spain (OCE-10-20), and the Bureau of Medicine (BUMED, NRD1170). The animals were trained to either slide onto a ledge or remain stationary inside the water where they rested voluntarily for up to 3 min, during which optical properties were measured. The animals had free access to the pool at any time and no restraints were used to hold animals in place, thus animals could leave the measurement site at any time. No sign of discomfort was noticed in the animal’s behavior while they were exposed to the measurement devices.

An FD-NIRS system (Imagent 2.0, ISS, Inc., Champaign, Illinois, United States) was used to measure $\mu_{a}$ and $\mu_{s}^{\prime }$ at seven different wavelengths in the near-infrared range between 730 and 830 nm. An optical probe with source and detector fibers spaced at 9, 18, 27, and 36 mm was placed on the dolphin’s skin between the pectoral fins on the chest. Light intensity was measured with optical fibers connected to four individual photo-multiplier tubes. Given the source–detector distances, the penetration depth of the light was not expected to exceed the ∼20  mm deep blubber layer. Therefore, the measured optical properties are assumed to represent only a combination of blubber and skin pigmentation. The light was modulated at 130 MHz to enable the calculation of phase delays between the source and detected light, alternating current (AC) component, and direct current (DC) component.

Using the multi-distance approach for optical property calculation,^25^^,^^26^ we determined the average $\mu_{a}$ and $\mu_{s}^{\prime }$ across the ∼2-min measurement period. We recorded a total of 117 measurements on 18 animals, in which only the 51 chest measurements were considered for this work due to a greater similarity in pigmentation across animals in this location. Every measurement was inspected for signal quality by ensuring that the modulation percentage mod%=AC/DC was >20%, no motion artifacts were observed, and no detector was saturated. Individual time frames and data channels showing data quality deficiencies were removed. In the end, a total of 41 measurements across 10 animals (2 Valencia + 2 Las Vegas + 6 Hawaii) were included in this study.

Individual time averages of $\mu_{a}$ and $\mu_{s}^{\prime }$ were calculated for each wavelength and measurement. The median across all measurements was calculated to estimate the skin and blubber optical properties. The median excluded any outlier values exceeding the interquartile range by more than 1.5 times the interquartile range.

In addition, for a subset of animals, broadband absorbance spectra were measured with a photo-spectrometer (FLAME-T-VIS-NIR-ES, Ocean Insight, Inc., Orlando, Florida, USA) and a tungsten halogen light source (HL-2000-HP-FHSA, Ocean Insight, Inc., Orlando, Florida, United States). Source and detector fibers were placed on the skin close to the location of the FD-NIRS measurements with a source–detector distance of 15 to 18 mm. With integration times of 4 to 20 s, depending on the environmental conditions and absorbance of the dolphin tissue, the received light was accumulated and saved over a wavelength range of 350 to 1000 nm. A total of 51 trials (32 on the chest) on a total of 6 animals were recorded. The measurement duration of a trial was 2 min, comparable to FD-NIRS measurements, however, the sampling rate depended on the integration time and the number of samples is therefore inconsistent. After rejection of saturated samples, often due to motion or influence of ambient light occurring within the long integration times, all samples were smoothed using a boxcar of 15 indices, corresponding to about 4.2 nm wavelength intervals, and averaged. The average absorbance across all remaining 18 measurements was calculated. To obtain absolute $\mu_{a}$, the reflectance in the wavelength range of 700 to 900 nm was fitted to the FD-NIRS-measured $\mu_{a}$ of the blubber tissue.

We used a least square fitting algorithm (“lsqcurvefit,” MATLAB 2022a, The MathWorks, Inc., Natick, Massachusetts, United States) to estimate oxygenated and deoxygenated hemoglobin concentrations, water (W), melanin (M), and fat (F) based on the spectrogram between 700 and 900 nm. For hemoglobin concentrations, the weights represented the blood volume fraction (B) relative to whole blood and the tissue saturation with oxygenated hemoglobin (S) in accordance with the nomenclature in Ref. 23. Hemoglobin concentrations in whole blood were assumed to be 150  g/L. A lower bound of 20% fat and 10% water was set to prevent fitting to local minima not representing blubber histology. The scattering coefficient follows a power law $\mu_{s}^{\prime }=a\lambda^{-b}$, where λ is the wavelengths of light and a and b are coefficients that were fitted to match the $\mu_{s}^{\prime }$ measured by the FD-NIRS system.

The optical properties for brain, bone, and muscle tissue were taken from the literature. Specifically, $\mu_{a}$ and $\mu_{s}^{\prime }$ were taken from Ref. 24. Using the tissue specific concentration values from Ref. 24, the overall tissue absorption can be calculated through weighted summation of $\mu_{a,\text{absorber}}$.^27^^,^^28^ Thus the total tissue specific absorption coefficient is defined as

$$\mu_{a}=B(S)\mu_{a,\mathrm{HbO}}+B(1-S)\mu_{a,\mathrm{HbR}}+W\mu_{a,\text{water}}+M\mu_{a,\text{melanin}}+F\mu_{a,\text{fat}}.$$

The scattering coefficient over wavelength was reconstructed using the power law coefficients provided by Jacques.^24^

Finally, $r_{i}$ were taken from the literature with brain^24^ and muscle^29^ $r_{i}=1.37$, blubber^30^ with a higher lipid concentration was set to $r_{i}=1.45$, and bone^31^ $r_{i}=1.55$.

### 2.2 Finite-Element Model

**Fig. 1:**
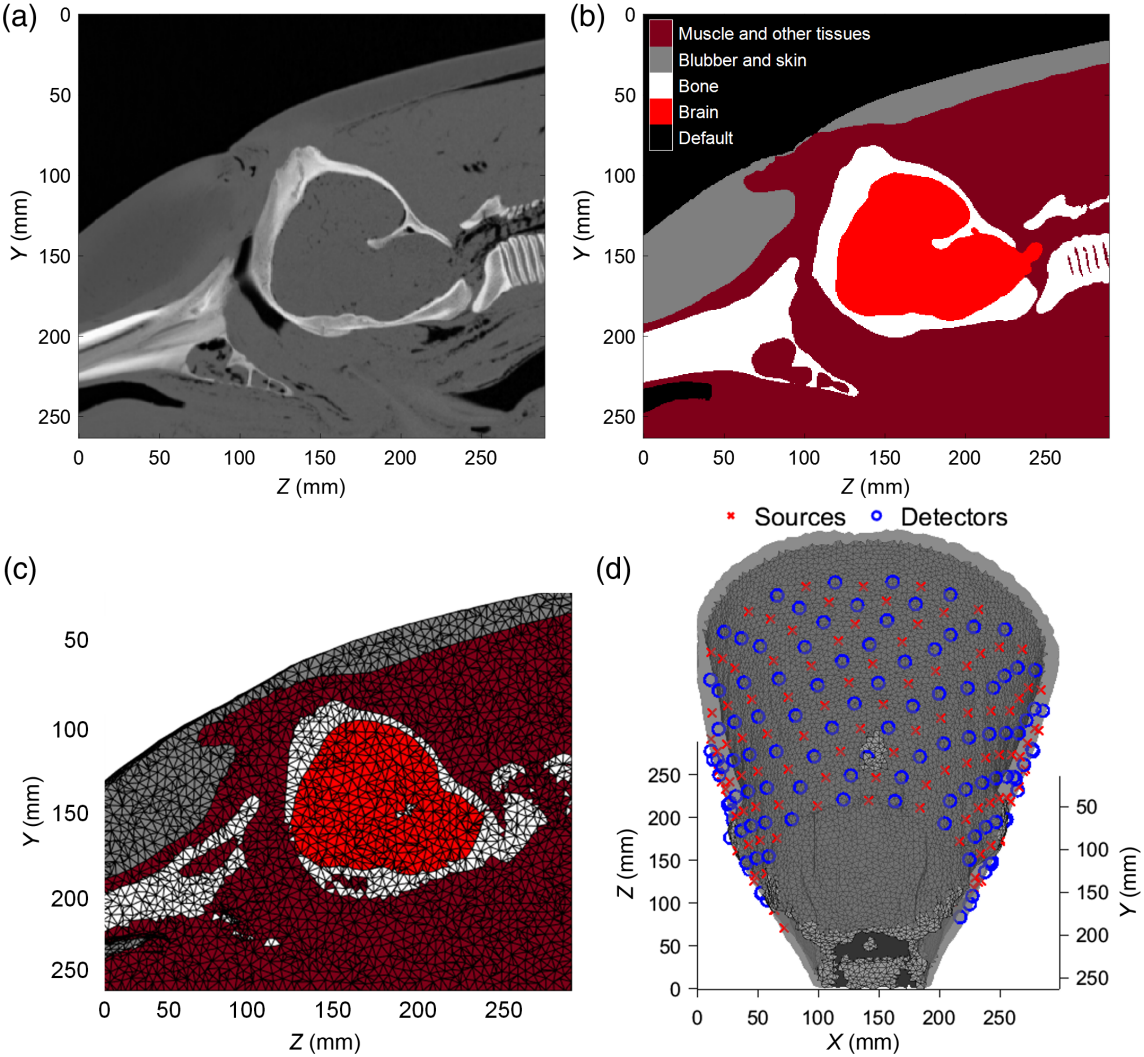
Mesh generation. (a) Sagittal plane cross-section of the CT scan used to generate the model. (b) Manually assigned tissue labels for the cross-section shown in (a). (c) Mesh generated from the tissue labels: blubber and skin (gray), muscle, and other tissues layer (dark red), bone (white), and the brain (light red). Black lines are the edges of tetrahedrons inside the mesh. (d) The same mesh as in (c) in non-opaque grayscale. Blue circles show the location of detectors, and red crosses show source locations.

A 3D head model was created based on a computed-tomographic (CT) scan [Fig. 1(a)] of a stranded striped dolphin (*Stenella coeruleoalba*), provided by the Fundación Oceanogràfic de la Comunitat Valenciana, Valencia, Spain. The 3D CT scan contained the full circumference of the dolphin from the tip of the rostrum to the third thoracic vertebra. Each voxel had a size of $0.586\times 0.586\times 0.7\;{\mathrm{mm}}^{3}$. The CT scan was segmented by hand using the image processing and segmentation program 3D Slicer.^32^^,^^33^ After segmentation, a 3D image (512×450×414  voxels) was saved containing the segmented tissues separated into five categories: (1) brain, (2) bone, (3) blubber and skin, and (4) muscle and other tissues, while the background was given a default value of 0—air [Fig. 1(b)]. No voxel of the CT scan was left unassigned, and no air was assigned to a voxel inside the volume.

MATLAB R2022a (The MathWorks, Inc., Natick, Massachusetts, United States) was used for processing, and the image was loaded using Fieldtrip^34^ function “ft_read_mri.”^34^ The segmented image was converted into a tetrahedral mesh using the Computational Geometry Algorithms Library’s 3D triangulation algorithm.^35^^,^^36^ The generated 82,512 nodes created 467,648 tetrahedrons [Figs. 1(c) and 1(d)]. The average tetrahedral volume was determined to be $24.97\;{\mathrm{mm}}^{3}\pm 9.47\;{\mathrm{mm}}^{3}$ (mean ± standard deviation). Every node inside the generated mesh was assigned $\mu_{a}$, diffusion coefficient $\kappa =1/3(\mu_{a}+\mu_{s}^{\prime })$, and $r_{i}$.

### 2.3 Photon Simulation

The diffusion equation describing the propagation of light in scattering media was solved at every node in the mesh to calculate the fluence rate of light at every point, using the NIRFASTer toolbox.^37^^–^^39^ The light sources and detectors were placed on the animal by finding the edge between dolphin skin and surrounding air in every labeled slice (XY images along the Z axis). Along the Z axis, 11 equidistant slices were chosen for optode placement, as seen in Fig. 1(d). Along the skin, 21 radially equidistant optodes were placed on every slice. After removing optodes to ensure that no pair fell within 10 mm of one another, a total of 222 optodes were alternated in sources and detectors, leading to an equal number of 111 sources and 111 detectors. Every source–detector pair within 50 to 120 mm of each other created a data channel. The resulting 2746 channels created a dense grid around the head.

Using the NIRFASTer toolbox,^37^^–^^39^ the forward model was calculated for continuous wave (CW) NIRS. The light projection was calculated for every source optode and the detector amplitude for every source–detector combination, or channel, given by the fluence rate at the detector position on the surface of the mesh assuming that only one source is active at a time. All fluence rates and amplitudes were computed relative to the input intensity of $1\;{\mathrm{mm}}^{-2}\;s^{-1}$. The optical properties of all tissues were reassigned, and the calculation was repeated for all wavelengths in the target range of 600 to 1700 nm, in 50 nm intervals.

After the forward modeling, the Jacobian matrix was derived.^40^ The Jacobian J (mm) is a sensitivity matrix that quantifies the fluence rate change in the detector given an absorption change (of $1\;{\mathrm{mm}}^{-1}$) in every node in the mesh for a specific source and detector combination, i.e.,

$$J=\partial \Phi /\partial \mu_{a}.$$

Thus J is the partial derivative of the fluence rate ∂Φ for a change in absorption coefficient $\partial \mu_{a}$. The influence of the change in scattering is assumed to be negligible under the assumptions of a CW illumination.

### 2.4 Quantitative Comparison and Statistics

To determine optimal source and detector positions, distances, and wavelengths, the following sensitivity measurements were calculated for wavelengths ranging from 600 to 1700 nm, in steps of 50 nm, varying the optical properties of the modeled tissue. To calculate the sensitivity to brain or muscle tissue, we utilized separate metrics: (1) Tissue sensitivity (TS) was calculated as the difference between simulated amplitudes before and after a local increase in oxygenated hemoglobin inside the target tissue. (2) Depth sensitivity based on the channel-specific sensitivity matrix J. (3) Depth sensitivity based on a flatfield change in absorption, set to 1% across the entire mesh. This analysis utilizes back projection to find the location of absorption coefficient change in the mesh and is performed within groups of channels with similar source–detector distance. (4) Light power inside a simulated large detector covering a $100\;{\mathrm{mm}}^{2}$.

#### 2.4.1 Tissue sensitivity

For the TS measurement, we ran a separate forward model, in which the hemoglobin content of the target tissue, here the brain, was increased by 10%, i.e., $B_{\text{brain},\text{active}}=1.1\cdot B_{\text{brain}}$. We calculated the TS as the difference between the two forward models, such that

$$\mathrm{TS}=\log_{10}(\Phi_{0})-\log_{10}(\Phi_{\text{active}})=\log_{10}(\frac{\Phi_{0}}{\Phi_{\text{active}}}),$$

where $\Phi_{0}$ is the fluence rate measured at the detector for every channel before the brain activation, and $\Phi_{\text{active}}$ is the fluence rate during activation. To ensure no channel has a uniquely and unrealistically high sensitivity to the target tissue and reduce the effect of numerical inaccuracies and poor mesh quality, we averaged all following sensitivity and intensity metrics across the highest 1% TS.

#### 2.4.2 Depth sensitivity: Jacobian

The Jacobian or sensitivity matrix shows the influence of brain tissue to measured light intensity. We therefore looked at the highest absolute value of the Jacobian inside the brain sub-mesh, relative to the highest absolute value of the Jacobian in the entire mesh. This metric indicates the total sensitivity of a specific channel to the target tissue.

#### 2.4.3 Depth sensitivity: flatfield

The flatfield analysis of depth sensitivity was based on work published by Dehghani et al.^38^ A flatfield, or uniform, increase of 1% in absorption is used to probe the influence of every point in the mesh on a given cluster of channels by

$$\partial \Phi =J\partial \mu_{a},$$

where $\partial \mu_{a}=\mu_{a}*0.01$ is the change in absorption and ∂Φ is the resulting change in fluence rate at the detector. Note that this inverse model is a tomographic approach to determine the depth in which the highest absorption change occurs. We compared source–detector distances by binning all channels into seven 10-mm-wide groups of source–detector distances between 50 and 120 mm. Using the back projection of the light propagation model, which calculates $\partial \mu_{a}$ for every node in the mesh based on measured detector intensities ∂Φ (here calculated as a flatfield), we answer the question of where in the mesh a change in absorption occurred. The back projection requires an inversion of J, defined according to Dehghani et al.^38^ and Culver et al.^40^ using the Moore–Penrose pseudoinverse^41^ as

$$L^{-1}{\overset{˜}{J}}^{T}{\left(\overset{˜}{J}{\overset{˜}{\;J}}^{T}+I\right)}^{-1}\partial \overset{˜}{\Phi }=\partial \mu_{a},$$

where λ is the Tikhonov regularization parameter applied to the identity matrix I. Further,

J˜=J^M−1,J^=JL−1,∂Φ˜=∂ΦM−1.

The regularization of the inversion algorithm is done in two parts, L is a depth-dependent spatial regularization defined as

$$L=\sqrt{\mathrm{diag}(J^{T}J)+\alpha },$$

with

$$\alpha =10^{-2}\;\max\;\mathrm{diag}(J^{T}J),$$

and M is a spatial regularization defined as

M=diag(J^J^T)+β,

with

β=10−2 max diag(J^J^T).

#### 2.4.4 Large detector placement

To determine if a detector can measure a subtle change in absorption, given a large source–detector-distance, we simulated placing large detectors with a total area of $A=100\;{\mathrm{mm}}^{2}$ onto the mesh’s surface. We then compared the fluence rate measured in these detectors before and during a simulated activation by increasing the hemoglobin content inside the brain by 10%. The detector size was simulated by placing a square grid of 51×51 simulated sub-detectors (N=2601) around the original detector location. The distance between sub-detectors was set to 0.2 mm. All sub-detectors amplitudes were calculated as described in 2.3. The total large detector fluence rate, or power, summed over the detector area was then approximated with

$$P=\frac{A}{N}\overset{N}{\underset{n=1}{\sum }}\Phi_{n}.$$

A detector must achieve a high dynamic range to not saturate at the maximum expected power $P_{\max}=\underset{N\;}{\max}[P_{0},P_{\text{active}}]$, while also being sensitive to the subtle difference in fluence rate between the activated brain and the brain at rest $\Delta P=P_{0}-P_{\text{active}}$. A small range between $P_{\max}$ and ΔP is therefore desirable for real-life detector selection.

### 2.5 Juvenile 3D Model

All analysis was repeated for a “juvenile” animal model, in which all dimensions of the model were shrunk by 25%. Although the number of nodes and elements in the mesh is unchanged, the average tetrahedral volume decreased to $10.71\pm 3.81\;{\mathrm{mm}}^{3}$ (mean ± standard deviation). The same amount of optodes on a smaller mesh resulted in a greater overlap within the defined 50 to 120 mm source–detector separation, totaling 4214 channels. All data post-processing and statistical analysis were repeated for the reduced mesh size and compared to the normal sized “adult” model.

## 3 Results

**Fig. 2:**
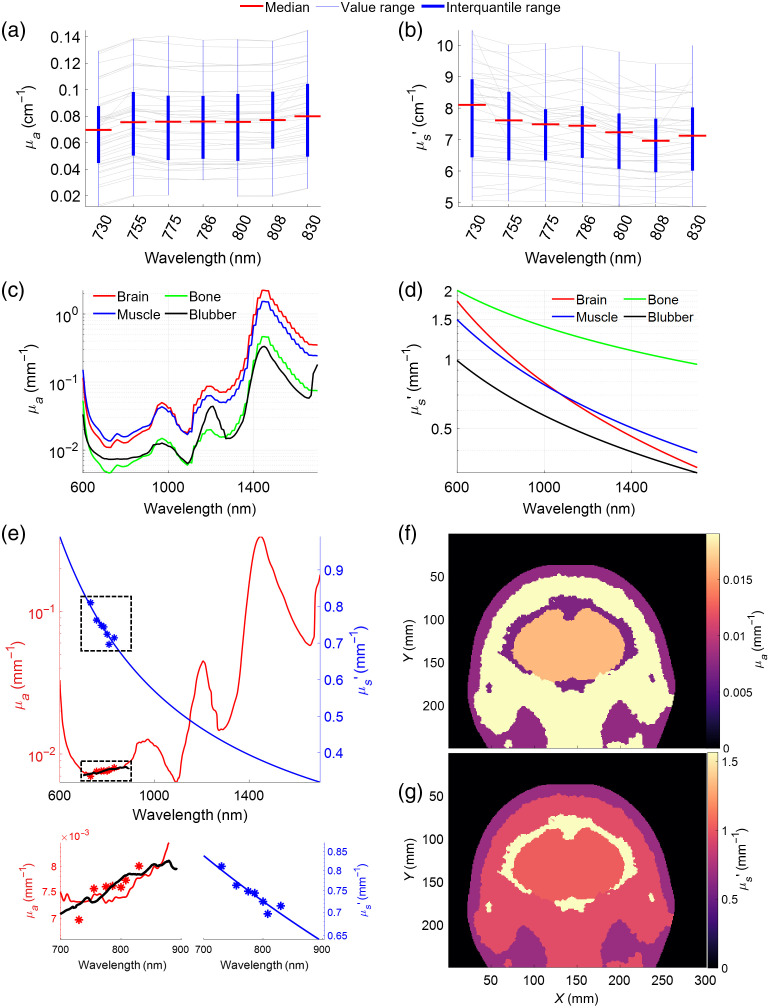
Optical properties taken from the literature and measured from bottlenose dolphins. (a) Measured absorption coefficient $\mu_{a}$. (b) Reduced scattering coefficient $\mu_{s}^{\prime }$. The gray lines show artifact-free measurements included in the analysis; red lines indicate the median after outlier rejection based on a maximum distance of 1.5 interquartile range from the median. (c) Absorption coefficient for brain, muscle, bone, and blubber tissue as assigned to the model. (d) Scattering coefficients assigned to all tissues in the model. (e) Results from fitting the absorption coefficients [graphs (a) and (b)] to the measured data (asterisks) to derive $\mu_{a}$ (red) and $\mu_{s}^{\prime }$ (blue). The black line shows the fitted absorbance from the broadband photo-spectrometer. Dashed boxes are enlarged below for a wavelengths range of 700 to 900 nm. (f), (g) A cross-section of the dolphin with optical properties at 850 nm.

Optical properties measured from the chest of 10 voluntarily participating Atlantic bottlenose dolphins in three different professional care facilities were averaged to obtain a general blubber and skin $\mu_{a}$ [Fig. 2(a)] and $\mu_{s}^{\prime }$ [Fig. 2(b)]. The measured absorption properties of the blubber tissue were lower than highly perfused brain and muscle tissues, but at some wavelengths higher than bone optical properties taken from the literature [Fig. 2(c), black line]. The scattering properties measured with the FD-NIRS device were, overall, decreased with wavelength, and when compared to other tissues [Fig. 2(d)] were overall low. The fitting results for the measured blubber and skin layer are shown in Fig. 2(e), where the black line shows the broadband photo-spectrometer measured absorbance after it had been fitted to the FD-NIRS measured $\mu_{a}$ (red asterisk). The least squares fit of tissue components then generated the red line. The weights for the fitting were determined to be B=0.006, S=0.98, W=0.1, M=0.00002, and F=0.2. Similarly, the blue asterisks show the measured $\mu_{s}^{\prime }$, which were used to fit the blue line according to the power law. The scaling factor and exponent to the scattering fit were determined to be a=1546 and b=0.8038. At a typical NIRS operation wavelengths of 850 nm, the muscles and brain are the most absorbing tissues [Fig. 2(f)], whereas the bone tissue is the most scattering component [Fig. 2(g)].

**Fig. 3:**
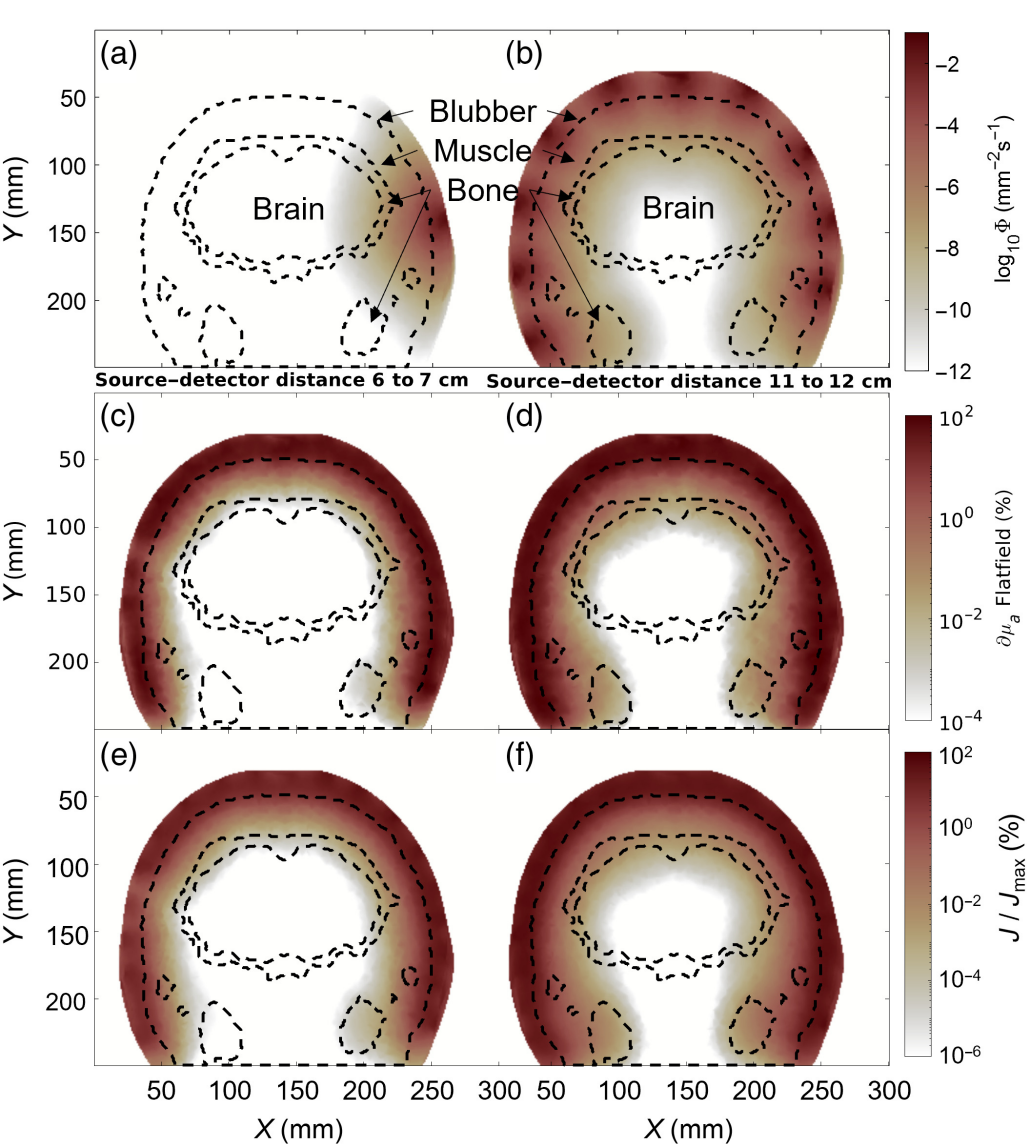
Adult dolphin sensitivity metrics. (a) The fluence rate of a single source on a coronal cross-section. Border lines of different tissues are shown in black dashed lines. (b) The full array of 10 sources placed around the mesh at this coronal slice. (c) Flatfield reconstruction for all channels with a source–detector separation between 60 and 70 mm as a percentage of the maximum value across the mesh. (d) Similar to (c), with a source–detector separation between 110 and 120 mm. (e) Summation of all sensitivity matrices (J) calculated for channels of 60 to 70 mm source–detector separation as a percentage of the maximum sensitivity ($J_{\max}$). (f) Similar to (e), for source–detector distances between 110 and 120 mm.

To fully understand the requirements for dolphin fNIRS measurements, we observed how light travels inside the dolphin’s body. We found that given a light source fluence rate of $1\;{\mathrm{mm}}^{-2}\;s^{-1}$, the light fluence rate decreased rapidly [see Figs. 3(a) and 3(b)], with a single light source losing as much as eight orders of magnitude in fluence rate before reaching the brain cortex. A circular arrangement of light sources can evenly illuminate the brain cortex for a full coverage of ±90  deg from the midsagittal plane [Fig. 3(b)]. To find the depth and tissue of most influence to the signal, we calculated the flatfield metric. At 60 to 70 mm source–detector separation, blubber tissue was the most sensitive area [Fig. 3(c)]. With a wider source–detector separation (e.g., 110 to 120 mm), we see the strongest influence on the measured light intensities originate from the border of blubber to muscle tissue layer [Fig. 3(d)]. To see how strong the contribution of the brain tissue is, we looked at the sensitivity matrix (J).

**Fig. 4:**
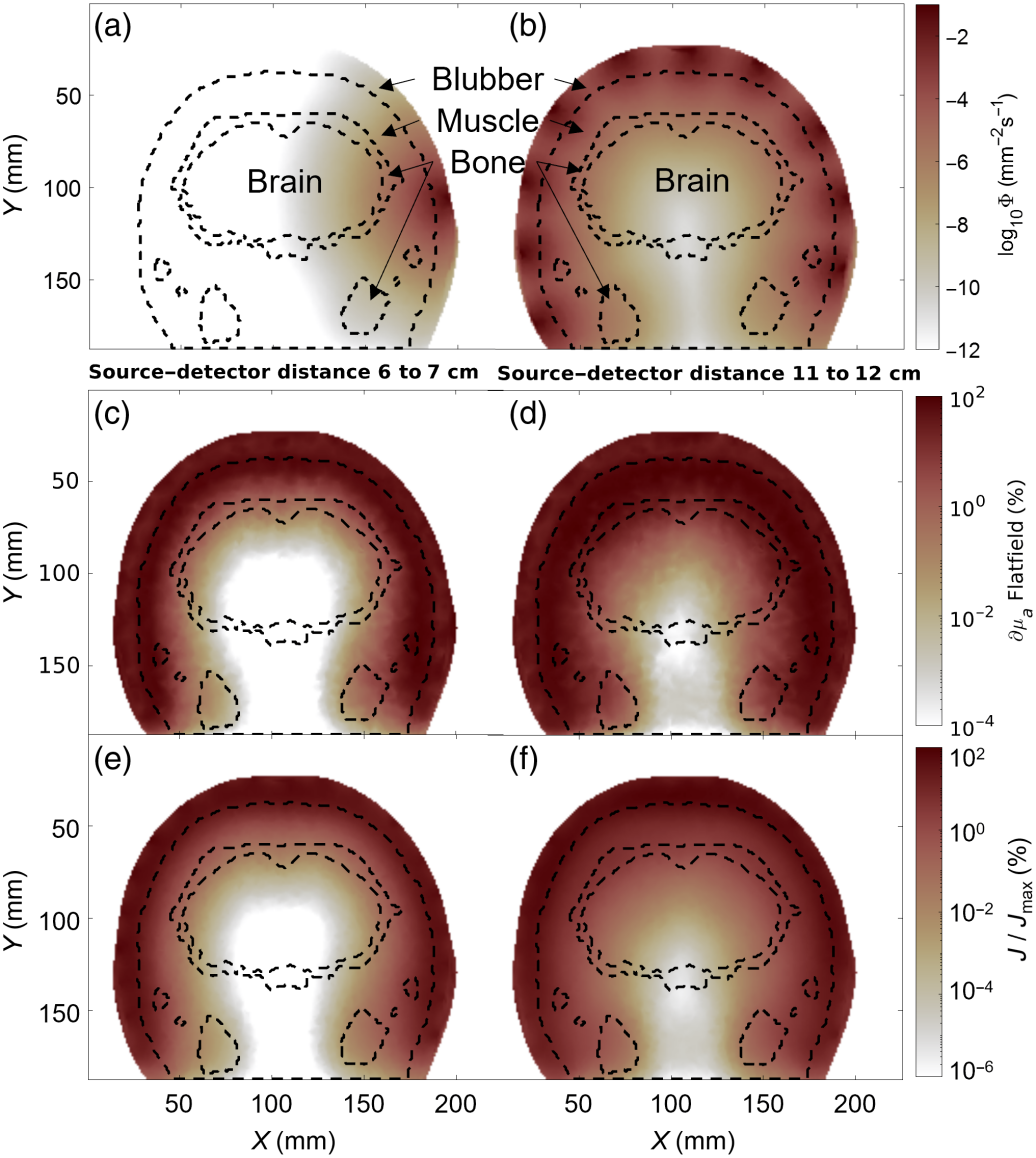
Juvenile dolphin (25% smaller than adult) sensitivity metrics. (a) The fluence rate of a single source on a coronal cross-section. Border lines of different tissues are shown in black dashed lines. (b) The full array of 10 sources placed around the mesh at this coronal slice. (c) Flatfield reconstruction for all channels with a source–detector separation between 60 and 70 mm as a percentage of the maximum value across the mesh. (d) Similar to (c), with a source–detector separation between 110 and 120 mm. (e) Summation of all sensitivity matrices (J) calculated for channels of 60 to 70 mm source–detector separation as a percentage of the maximum sensitivity ($J_{\max}$). (f) Similar to (e), for source–detector distances between 110 and 120 mm.

Figures 3(e) and 3(f) illustrate the sensitivity as a percentage of the strongest sensitivity in the entire mesh. At a 60 to 70 mm optode separation, we achieve a sensitivity of <0.001%. A larger source–detector distance can increase the sensitivity to the brain to about 0.01% [Fig. 3(f)]. The sensitivity increases when simulating a juvenile dolphin—shrinking the mesh size by 25%. Fluence rates in the brain are $>10^{-7}\;{\mathrm{mm}}^{-2}\;s^{-1}$ [Figs. 4(a) and 4(b)], with an increased sensitivity to deeper layers of the muscle tissue, where the maximum sensitivity borders on the skull [Fig. 4(d)]. The Jacobian confirms these improvements, with a sensitivity of 0.1% in the shorter source–detector distance [Fig. 4(e)] and a sensitivity of >1% with long source–detector distances [Fig. 4(f)].

**Fig. 5:**
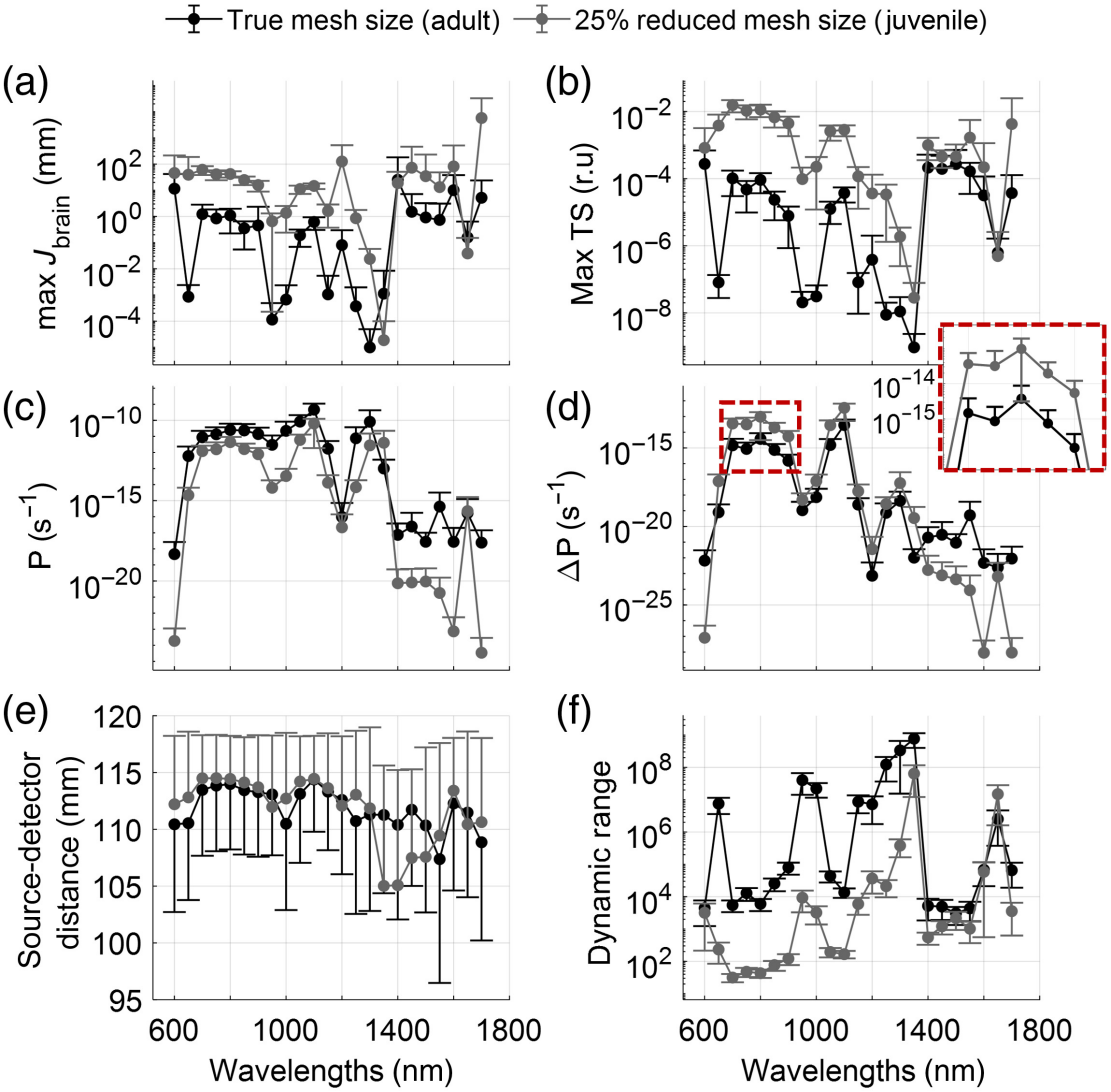
Quantitative comparisons across wavelengths and model size. Dots represent the average across the 1% of channels with the largest TS values. Error bars represent the standard deviation. Black lines show model results for an average-sized adult dolphin; gray shows results from a juvenile model, 25% smaller than the adult. (a) Jacobian values inside the brain tissue; (b) TS index; and (c) power as measured by a $100\;{\mathrm{mm}}^{2}$ detector relative to an input fluence rate of $1\;{\mathrm{mm}}^{-2}\;s^{-1}$. (d) Difference in light power between a brain at rest and an active brain, simulated by increasing the hemoglobin concentration inside the brain mesh by 10%. The dashed line indicates the area shown in greater detail in the inset at the top right of the panel. (e) Source–detector distances for the selected 1% best channels. (f) Dynamic range, defined as ΔP/P, showing the required dynamic range that the detector must achieve.

We compared the adult and juvenile models for different quantitative measurements. The most sensitive 1% of channels was found based on the TS metric. When comparing a mean across the 1% most sensitive channels to brain tissue based either on the maximum J inside the brain mesh or the maximum TS index, we found a more than two orders of magnitude increase in sensitivity [Figs. 5(a) and 5(b)] for the juvenile model compared to the adult model. The most sensitive wavelength is between 700 and 900 nm in the adult model and 1400 and 1600 nm in the juvenile model. Because this sensitivity metric does not consider the total amount of light reaching the detector, we also looked at the detector amplitudes of the most sensitive channel. The total measured power in a $100\;{\mathrm{mm}}^{2}$ detector was calculated to be $∼10^{-10}$ with respect to a light source fluence rate of $1\;{\mathrm{mm}}^{-2}\;s^{-1}$ at 1100 nm and slightly lower at 700 to 900 nm. The power decreases strongly as wavelength increases beyond 1100 nm [Fig. 5(c)]. The detector power difference ΔP between the activated brain and the brain at rest showed an intensity difference of $10^{-15}\;s^{-1}$ at 800 to 900 nm and 1050 to 1100 nm. The juvenile model, while receiving less absolute light intensities, showed a ΔI of $10^{-14}\;s^{-1}$ at 800 to 900 nm, and thus an order of magnitude improvement [Fig. 5(d)]. Both juvenile and adult meshes showed better sensitivity at the longest source–detector distance [Fig. 5(e)]. Another practical consideration for selection of a detector capable of measuring the brain activity is the dynamic range of intensities it must cover. The juvenile dolphin model had greater brain sensitivity with slightly lower absolute fluence rates and thus requires a smaller dynamic range. We estimated that the required dynamic range was about $10^{4}$ for wavelengths in the ranges of 800 to 900 nm and 1050 to 1100 nm in an adult dolphin model; it was about $10^{2}$ in the juvenile model [Fig. 5(f)].

**Fig. 6:**
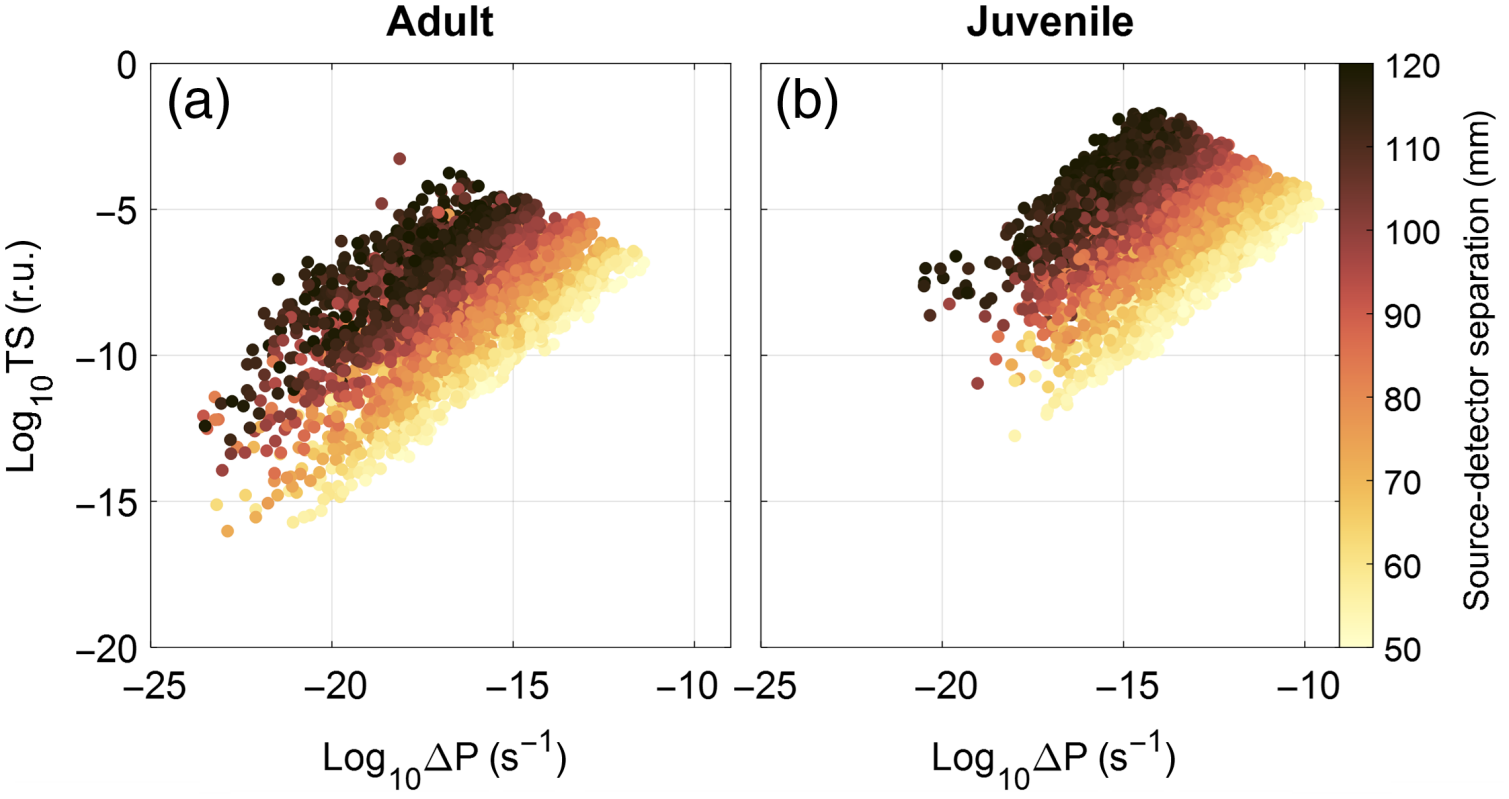
Sensitivity at 850 nm as a function of source–detector separation: (a) sensitivity in the adult dolphin mesh and (b) the reduced juvenile mesh results. The TS [in relative units (r.u.)] is plotted over the change of light power in the detector due to brain activation. Color coded is the source–detector separation.

The best source–detector distance can be found from the above results by considering one of the most sensitive wavelengths, 850 nm. For this wavelength, we plotted the sensitivity and optical power metrics as a function of optode separation. In both the adult and juvenile models, we found that the largest source–detector separations (110 to 120 mm) yield the highest sensitivity to the brain tissue; however, these source–detector distances show lower ΔP changes (see Fig. 6).

**Fig. 7:**
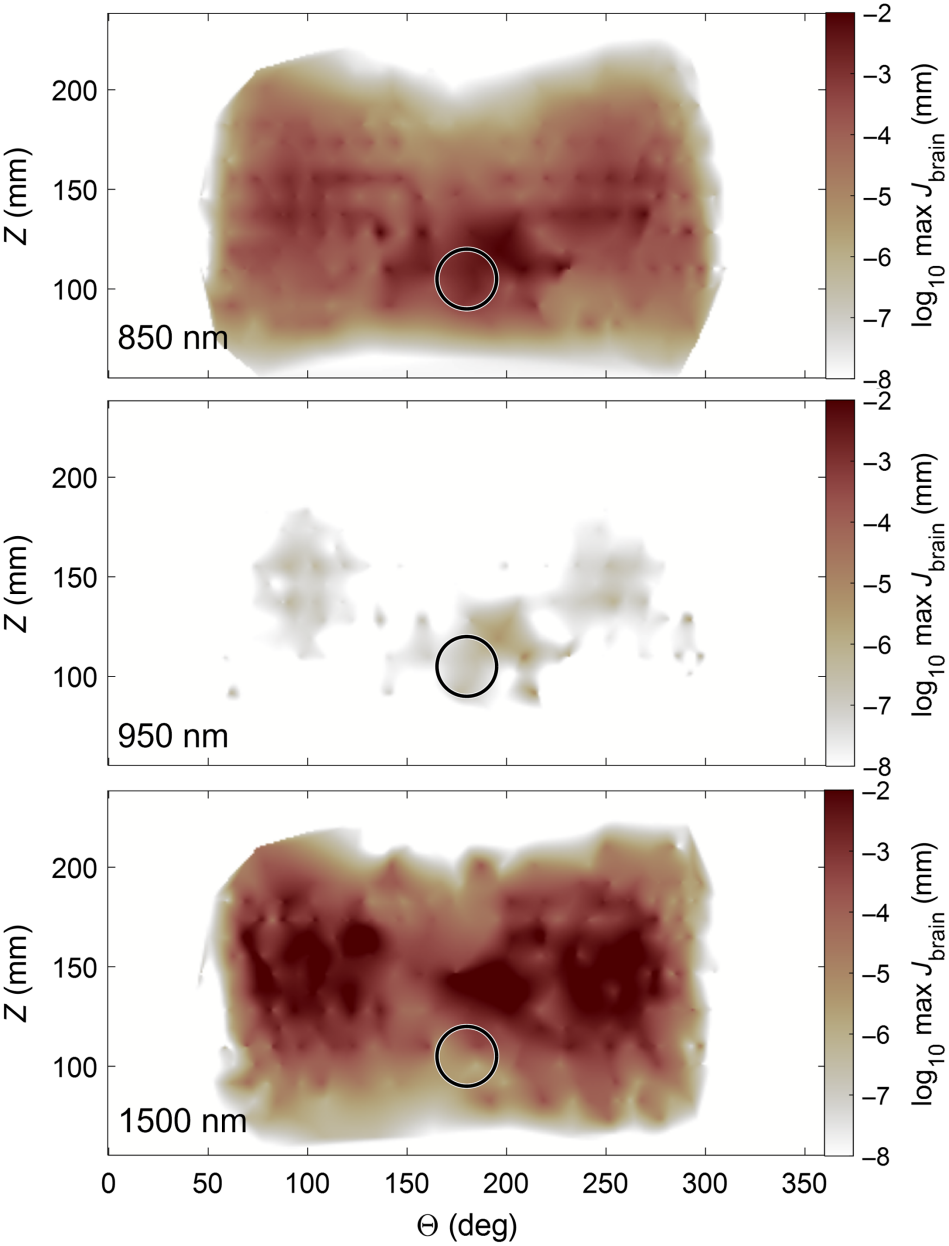
Brain-sensitive locations for optode placement. The maximum Jacobian value inside the brain mesh is used as a metric of brain sensitivity. The location on the map is based on the center point between source and detector of the individual channel, expressed in polar coordinates. Here the dolphin is facing downward, with the circle approximating the location of the blowhole. 180 deg is the midsagittal plane. The colors were interpolated across all channels and plotted for three example wavelengths, i.e., 850, 950, and 1500 nm.

Finally, to find the optimal location for optode placement, we plotted the maximum Jacobian inside the brain for every channel at the position halfway between the source and detector. The location most sensitive to changes in brain signal was located between 45 and 90 deg from the midsagittal plane, about 30 to 50 mm behind the blowhole (see Fig. 7).

**Fig. 8:**
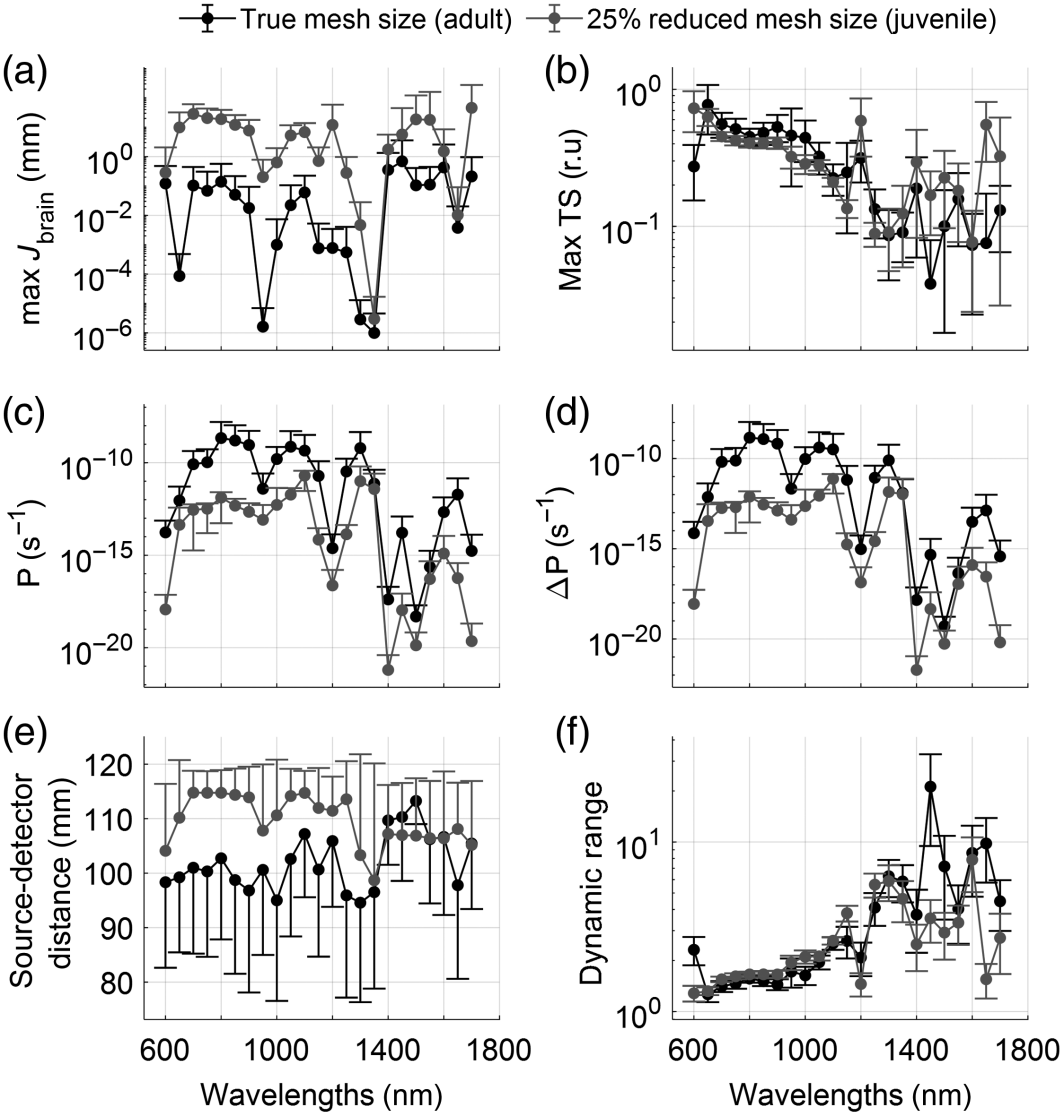
Quantitative comparisons across wavelengths and model size. Dots represent the average across the 1% of channels with the largest TS values. Error bars represent the standard deviation. Black lines show model results for an average-sized adult dolphin; gray shows results from a juvenile model, 25% smaller than the adult. (a) Jacobian values inside the muscle tissue. (b) Muscle TS index. (c) Power as measured by a $100\;{\mathrm{mm}}^{2}$ detector relative to an input fluence rate of $1\;{\mathrm{mm}}^{-2}\;s^{-1}$. (d) Difference in light power between a muscle at rest and a stronger perfused muscle, simulated by increasing the hemoglobin concentration inside the muscle tissue mesh by 10%. (e) Source–detector distances for the selected 1% best channels. (f) Dynamic range, defined as ΔP/P, showing the required dynamic range that the detector must achieve.

Interpretation of the data with respect to brain sensitivity has led to discussions on sensitivities of other tissues and the utility of NIRS as a measurement of physiological parameters, such as tissue saturation with oxygenated hemoglobin or heart rate dynamics. We thus investigated the sensitivity to muscle tissue by changing the hemoglobin concentration inside the muscle tissue by 10%, comparable to the brain activation in Fig. 5. A much higher sensitivity overall and dependency as a function of wavelength was found in the TS index [Fig. 8(b)]. Despite comparable absolute power measurements in the simulated detector [Fig. 8(c)], given similar source–detector separations, the change in power as a result of the total hemoglobin concentration increase was much greater than previously observed in the brain [Fig. 8(d)], leading to a greatly reduced dynamic range required to measure these signal [Fig. 8(f)].

## 4 Discussion

The main goal of this work was to determine the sensitivity of NIRS to functional activation in a dolphin’s brain within practical limits, to explore the potential utility of fNIRS to measure brain activity in medium sized cetaceans. We measured optical properties of dolphin blubber and combined it with literature values of optical properties from muscle, bone, and brain tissue. We simulated light travel through a 3D model of a striped dolphin and extracted brain sensitivity metrics. Overall, we found that the subcutaneous blubber layer and muscle layer absorption and the increased distance to the brain as compared to human fNIRS applications, makes functional brain measurements in dolphins challenging. We therefore investigated the influence of animal size, by simulating a juvenile animal through 3D mesh size reduction. We further laid out sensitivity metrics for muscle tissue to discuss the use of NIRS as a tool for animal healthcare and research. These findings are further discussed below.

We estimated that, as a percentage of the maximum value in the Jacobian matrix, sensitivity to brain tissue in adult bottlenose dolphin falls between 0.01% and 0.001% for source–detector separations of 110 to 120 mm [see Figs. 3(e) and 3(f)]. In contrast, for a human model,^38^ a brain sensitivity of >1% can be achieved with source–detector separations of <30  mm. Given anatomical constraints in adult dolphins, a source–detector separation of up to 120 mm cannot achieve a signal quality comparable to human models. However, we have shown some sensitivity to signals deriving from the brain (Figs. 3 and 5). In a setup with external stimuli or cognitive tasks, data quality like that achieved in human research would require a larger number of trials to increase the signal-to-noise ratio and average out physiological artifacts. The sensitivity is improved by an order of magnitude for a juvenile dolphin or a smaller dolphin species (simulated here with a 25% reduction in size). For such smaller animals, we can achieve comparable results to human fNIRS with a sensitivity close to or exceeding 1% at source–detector distances >60  mm (Fig. 4).

These sensitivity percentages assume a wavelength of light of 850 nm, which is one of the most sensitive wavelengths to brain tissue [Fig. 5(b)]. It is also a wavelength used in many commercially available NIRS devices for use in humans. Yet it is not the only wavelength that is suitable for fNIRS applications in dolphins. High sensitivity to brain tissue, as measured by the TS index, was also found for wavelengths between 700 and 900 nm, 1050 and 1100 nm, and 1400 and 1550 nm [Fig. 5(b)], which is reflected in the sensitivity matrix [Fig. 5(a)]. Despite high sensitivity in the longer wavelength range, we see a steep decline in measured detector amplitudes for wavelength >1100  nm. This is likely due to the increase in water absorption, which is a major contributor in most tissues. Combining the TS to the brain with the returned light power, optimal wavelengths for imaging the brain are between 700 and 900 nm or 1050 and 1100 nm. The gap at 950 and 1000 nm is likely attributed to high fat absorption (see Fig. 2).

Selection of a suitable detector depends both on the minimum required light sensitivity to pick up brain activations and the largest expected light intensity, which together determine the required dynamic range of the detector. Increasing a sensor’s dynamic range tends to increase its cost, which is an important practical limitation. We found that the wavelengths that maximized the light received and achieving the highest brain TS also required a smaller dynamic range. The smallest dynamic range required in the adult model was $<10^{4}$; the juvenile model required a dynamic range two orders of magnitude smaller, i.e., $<10^{2}$ [see Fig. 5(f)]. This dynamic range was based on results from the 1% of channels that were most sensitive to signals originating from the brain according to the TS [Fig. 5(a)], with source–detector separations between 110 and 120 mm. Large source–detector separations are necessary for enough light to reach the brain but lead to low received light intensity and require sensors to have a large dynamic ranges. However, the optimal compromise across these constraints depends on the size of the animal as the dynamic range decreases by a factor of 100 and the light power difference to brain activation increases by a factor of 10 when imaging a juvenile dolphin [Fig. 5(d)].

The optimal source–detector location for fNIRS depends jointly on the amount of light received in the detector and the sensitivity of the received light to signals from the brain (Fig. 6). Selection of the optode separation has a tradeoff, and the optimal parameters vary with the size of the animal. For our adult dolphin model, a source–detector separation of >100  mm is within one order of magnitude of that of the most sensitive channel and can create a trade-off between received light intensity and source–detector separation. This distance will increase for larger animals and can be reduced for smaller ones.

In addition to wavelength and source–detector separation, we also explored how optode placement affected sensitivity. The Jacobian matrix can tell us about the sensitivity at a specific location, i.e., the contribution of a specific node inside the mesh to the measured light intensity in any channel. The highest sensitivity to brain tissue, according to the largest Jacobian values, was found in channels covering an area 30 to 50 mm behind the blowhole at 45 to 90 deg from the midsagittal line (Fig. 7). This location refers to the midpoint of the source–detector distance. Although the overall sensitivity changed with wavelength, the location of best sensitivity remained comparable.

Although brain fNIRS measurements appeared challenging with today’s commercial NIRS devices, physiological measurements of muscle and blubber tissue for behavioral research and veterinary purposes appear feasible. We have previously demonstrated the ability to measure cardiac pulsation and respiration from a bottlenose dolphin’s chest^13^ at separations <50  mm. Our simulation results confirm these findings. A source–detector distance of 60 mm shows a high sensitivity to the muscle layer underneath the blubber in both adult and juvenile dolphins, as shown by the signal percentage of the flatfield images [see Figs. 3(c) and 4(c), respectively]. The muscle sensitivity is increased greatly when comparing brain [Fig. 3(b)] to muscle tissue [Fig. 8(b)] within the same model. In fact, a 10% total hemoglobin concentration increase in the brain reveals a sensitivity more than three orders of magnitude lower than in muscle tissue. This trend is reflected in the dynamic range between activated and rested tissues.

Our model therefore suggests that muscle tissue measurements in adult dolphins are possible with commercially available NIRS devices, whereas brain fNIRS measurements will require highly sensitive, specialized equipment.

The models and assumptions used here represent our best good faith estimates of tissue properties and anatomical structures in dolphins; however, some caveats should be highlighted. To simulate how sensitive a NIRS measurement on the surface would be to hemodynamic signals originating from deeper tissues, we first needed to create a finite-element model and assign optical properties to different layers. The model created here is based on the CT scan [see Fig. 1(a)] of a stranded, deceased dolphin. In creating the segmentation, some tissue had to be interpolated. The segmentation was compared to information about dissected animals from the literature to ensure a realistic allocation of tissue groups, which at times required the interpolation of smaller air pockets created by the decay of connective tissues. These pockets were interpolated with surrounding muscle tissue.

The anatomical structure underlying all simulations is only based on a single individual. Subject variations as a function of age, sex, season, and location are beyond the scope of this work; however, they may play an important role in the interpretation of this data. For example, the thickness of the blubber layers can vary between summer and winter season. Additionally, a juvenile animal might have anatomical differences, which are not captured by simple miniaturization of an adult 3D model.

Each node of the mesh generated from the segmentation is a point in the finite-element model to which we assigned light absorption, scattering, and refraction properties. The assumed values of these parameters were taken from a review by Jacque,^24^ most of which came from terrestrial mammal tissue measurements. These optical properties were used for the brain, bone, and muscle layers. Light-absorbing tissue in terrestrial mammals may have a different composition from that in marine mammals. For instance, proteins like myoglobin tend to be more common in the muscle tissue of deep diving marine mammals than in terrestrial mammals.

Finally, we limited the source–detector separation to 120 mm. Further increasing the source–detector separation is theoretically possible to increase the brain sensitivity, but the returned light intensity would be further decreased. Additionally, a larger source–detector separation will decrease the spatial resolution of fNIRS and limit the purpose for observation of specific cortical activation. Although we have shown that brain imaging in dolphins with NIRS is in principle possible at 120 mm source–detector distance, further evaluation of practicality will be needed.

## 5 Conclusion

We have shown that with our assumptions, achieving brain fNIRS measurements in adult bottlenose dolphins will be challenging. Signal strengths of brain activations are close to the limit of what even a specialized maximum light power, maximum sensitivity NIRS device could theoretically achieve. Nonetheless, fNIRS might be possible in specific locations at source–detector distances >100  mm and at wavelength from 700 to 900 nm or 1100 to 1150 nm. We also found that smaller dolphins, such as juvenile animals or potentially a smaller species, can greatly improve the received signal from the brain. Our simulations also showed that in both adult and juvenile models, sensitivity to muscle tissue layers dominated measurements with shorter source–detector separations of 50 mm and likely below. Still, development of NIRS to study cerebral physiology in dolphins could yield a valuable tool in animal care and help unravel adaptations that allow these marine mammals to perform extreme breath-hold diving. Although muscle perfusion and oxygenation can be measured using off-the-shelf NIRS devices, functional brain measurements in dolphins will require development of specialized tools that deliver and sense greater amounts of light than in human fNIRS to support the large source–detector separations necessary to reach the brain.

## Code, Data, and Materials Availability

The work presented here utilizes the NIRFASTer toolbox, which is publicly available on GitHub: https://github.com/nirfaster/NIRFASTer.git. The 3D model, optical properties, and code required to reproduce the analysis discussed in this article will be made available under doi:10.24433/CO.6763388.v1.
